# Supplementary material for: Lack of Association between Serum Chitotriosidase Activity and Arterial Stiffness in Type 2 Diabetes without Cardiovascular Complications
Source: Int J Mol Sci. 2023 Oct 31;24(21):15809. doi: 10.3390/ijms242115809 (PMC10648693; doi:10.3390/ijms242115809)
Supplement: Supplementary file 1 [file ijms-24-15809-s001.zip › Supplementary_S1.pdf]

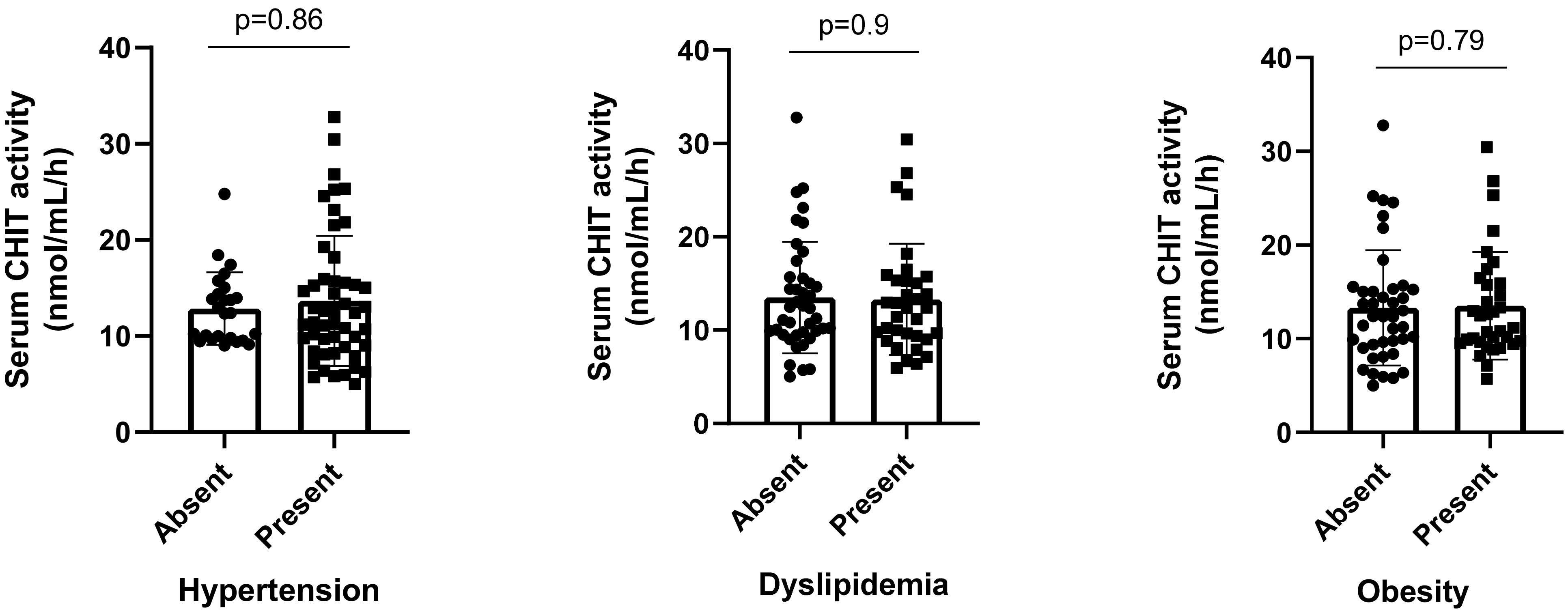

Supplementary Figure 1. Difference in CHIT activity stratifying the study population according to the presence of hypertension, dyslipidemia or obesity. *CHIT*, chitotriosidase.
